# Effects of aromatherapy with Matricaria chamomile essential oil on anxiety and hemodynamic indices in patients with acute coronary syndrome, 2021: a randomized controlled trial

**DOI:** 10.1186/s12906-023-04326-9

**Published:** 2024-01-03

**Authors:** Majid Pourshaikhian, Mohammad Taghi Moghadamnia, Ehsan Kazemnezhad Leyli, Zahra Shafiei Kisomi

**Affiliations:** 1 School of Nursing and Midwifery, Guilan University of Medical Sciences, Rasht, Iran; 2 School of Nursing and Midwifery, Qazvin University of Medical Sciences, Qazvin, Iran

**Keywords:** Aromatherapy, Matricaria chamomile, Acute coronary syndrome, Anxiety, Hemodynamic indices

## Abstract

**Background:**

Patients with Acute Coronary Syndrome (ACS) experience high levels of anxiety that may cause instability of hemodynamic indices, increased risk of ischemia, myocardial infarction and poor quality of life. Aromatherapy can affect patients’ anxiety levels and improve hemodynamic indices. This study aimed to evaluate the efficacy of aromatherapy on anxiety and hemodynamic indices in ACS patients.

**Methods:**

This study was a double-blind, randomized clinical trial conducted on 154 ACS patients. The participants were classified into two equal groups of intervention and placebo through the block randomization method. The data collection tools consisted of demographic information, a shortened 6-item version of the Spielberger questionnaire and a form of hemodynamic indices. For two consecutive nights, the intervention group inhaled 7 drops of the chamomile essential oil (%10) and the placebo group inhaled 7 drops of the sesame oil poured on a sterile cotton ball. The hemodynamic indices were collected half an hour before, one and four hours after the intervention until the next morning. The Spielberger questionnaire was completed once before the intervention and once after the end of the intervention, by the researcher through an interview. The number of heart rate (HR) was counted for a full minute. Also, the blood pressure (BP) of all the samples was measured by the researcher. Data analysis was done using Chi-square, paired t-test, and analysis of variance (ANOVA) in SPSS_22_.

**Results:**

The mean and standard deviation of the age of patients were 58/2 ± 11.6 and 59.7% of them were female. The results of ANOVA showed a significantly lower anxiety score as well as systolic blood pressure (SBP), diastolic blood pressure (DBP) and HR in the intervention group compared to those of the placebo group (*P* < 0.001). The decrease in anxiety score after the intervention, in the intervention and placebo groups was (5.2 ± 1.9) and (1 ± 1. 18) respectively. In the intervention group, the SBP and DBP after the intervention, was significant (*P* < 0.05). Also, the HR was significant (*P* < 0.001) after the intervention.

**Conclusions:**

Aromatherapy could reduce anxiety and improve hemodynamic indices in ACS patients.

**Trial registration:**

IRCT20080825001083N11.

## Background

Cardiovascular diseases cause death and disability more than any other disease and create financial costs in developed countries. Ischemic heart diseases (IHD) are the most prevalent, serious, chronic, and dangerous illnesses in the United States [1]. According to the World Health Organization (WHO), 17.9 million people died due to cardiovascular diseases in 2021, and these diseases account for 32% of all global deaths [2]. In Iran also these diseases lead to 90 thousand deaths yearly [3].

ACS is an emergency condition characterized by acute and sudden myocardial ischemia that can lead to myocardial infarction (MI) if not treated [4]. This syndrome includes a range of conditions including, unstable angina, non-ST elevation MI, and ST elevation MI. Patients may experience a combination of symptoms of chest pain, shortness of breath, indigestion, nausea, and anxiety caused by stimulation of the sympathetic nervous system [5]. About 50% of these patients report anxiety symptoms. It can be due to fear of death, fear of repeated stroke [4], separation from the family, unfamiliar environment, high noise, continuous light, sleep disturbance, pain and discomfort, lack of knowledge of diagnostic methods, treatment costs, concerns about self-care and getting back to work. Following anxiety, the amount of blood Catecholamines, Adrenocorticotropic Hormones, Prolactin, Cortisol, and Prostaglandin increases [6], as a result, the heart rate, heart contraction force and blood pressure increase, which leads to the heart’s high demand for oxygen and the blood supply to heart cells is disturbed [7]. Therefore, the risk of ischemia, and MI increases [8]. The main indices that can be suitable criteria for measuring the condition of ACS patients are breathing, pulse, blood pressure and temperature [7]. Since patients with MI who have anxiety are 2.5 times more exposed to ischemia, reducing anxiety in these patients is vital [9].

There are various strategies to reduce anxiety, including pharmacological and non-pharmacological treatments. Non-pharmacological treatments include complementary and alternative medicine (CAM), aromatherapy, massage therapy, etc. [10]. which can be used alone or in combination with other methods [11]. Nowadays there is a great desire to use these methods [12]. The advantages of CAM are cheapness, non-invasiveness, easy implementation, and the absence of chemical side effects [13, 14]. One of the methods of CAM is aromatherapy, which uses volatile and aromatic oils of plants to promote the level of physical, spiritual, mental [15] and physiological health [16, 17]. Today, aromatherapy is usually used to eliminate pain, promote relaxation, and relieve daily anxiety, depression [17] and stress [18]. Essential oils enter the body through the integumentary and olfactory systems and directly affect the brain [19]. Olfactory aromatherapy is able to optimize the mood or otherwise benefit the state of mind adversely affected by life factors and the subsequent effects of the illnesses like anxiety, depression and Inhalation of essential oils is a fast, convenient and safe method [20]. Smells affect the brain. The olfactory system is connected to the limbic system, which is the emotional control center—hence involved in controlling stress and hormone balance [21] and positively affect the blood pressure, heart rate, the tone of muscle [22]. In fact, odors are able to change the emotions in human [21].

One of the most widely used herbs in aromatherapy is chamomile, which using it has been declared safe by the American Food and Drug Administration [23]. Matricaria chamomilla, chamomile, is an herb belonging to the Asteraceae family. It contains many phytochemicals, such as alpha-bisabolol, bisabolol oxide, kamazolen, and flavonoid. Chamomile is known to exert an anti-inflammatory, antibacterial, and antifungal effect. It also has analgesic effects and repairs damaged tissues [24]. Chamomile is traditionally used in Iran due to its antipyretic effects, strengthening of the nervous and immune systems, sleep-inducing, relaxation and pain relief [25, 26]. Today, the use of chamomile in the treatment of gastrointestinal disorders [27], mastitis, neuralgia, convulsions [28], reducing anxiety, depression [29], pain and infection [30], skin inflammation, cough caused by bronchitis, fever, colds, treating wounds and burns has been approved and no complications have been mentioned for its consumption [25]. There is evidence of the existence of flavonoids with the same function as Benzodiazepine and Phytoestrogens in the chamomile plant, which seems to be able to show anti-anxiety effects [31–33]. Although chamomile has been widely introduced worldwide as an anti-anxiety agent, human research is limited, and it has always been suggested that more clinical trials be conducted to compare the efficacy of chamomile essential oil with conventional medications and determine its risk/benefit [34, 35]. Although anti-anxiety drugs are not the only way to control anxiety, and due to the side effects of chemical drugs and the difference in patients’ responses to them, it is crucial to use non-pharmacological methods to reduce patients’ anxiety, it has been observed that nurses pay less attention to non-drug treatment methods compared to drug methods [36]. On the one hand, reducing patients’ anxiety and fear is one of the most important nursing measures [4]. On the other hand, nurses spend a lot of time with patients [36] and the monitoring and controlling of hemodynamic changes is one of the nursing care plans, and the stability of these indices is important. Therefore, the present study aimed to determine the efficacy of aromatherapy with chamomile essential oil on anxiety and hemodynamic indices of ACS patients.

### Hypotheses

Aromatherapy with chamomile essential oil is effective in reducing anxiety in ACS patients hospitalized in male and female heart departments. Besides, aromatherapy with chamomile essential oil is effective in improving hemodynamic indices (Systolic Blood Pressure, Diastolic Blood Pressure and Heart Rate) of ACS patients hospitalized in heart departments.

## Materials and methods

### Study design

This was a parallel, randomized, double-blind clinical trial study. The study population included patients diagnosed with ACS and hospitalized in male and female heart departments of Booali Sina Teaching Hospital of Qazvin University of Medical Sciences in 2021.

Sampling and randomization.

**Fig. 1:**
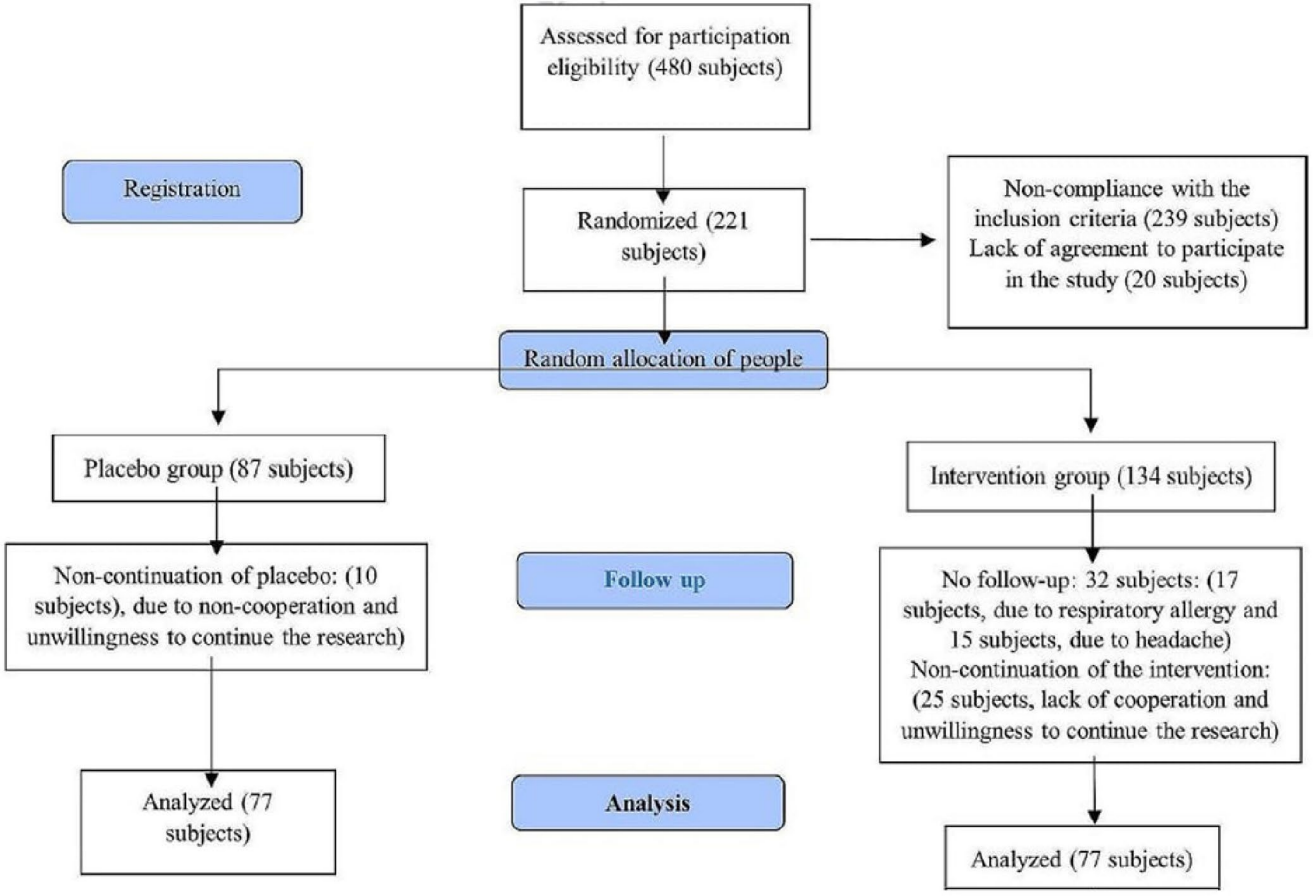
The process of the CONSORT 2010

The subjects included 154 ACS patients who were selected using the cluster random sampling method. They entered the study in two groups and the sample size was calculated to be 77 subjects for both groups, with a test power of 80% and a significance level of 95% (Fig. 1).

To calculate the sample size, we used the results of a study entitled “Investigating the effect of chamomile scent on the anxiety of endoscopy candidate patients” [23]. The mean of the anxiety score after the intervention of the patients in both groups in the mentioned study was 39.88 and 35.89, respectively. Therefore, the required sample size in each group was calculated as follows:

$$\text{n} \cong 77\frac{(1.96+0.84)^{2}(8.4+9.3)^{2}}{\left(39.8{8}_{-}35.89\right)^{2}}$$

The effect size, according to the mentioned sampling formula based on the difference of the anxiety score of the two groups, and the standard deviation of the anxiety score is equal to 0.5 (on average). The recruitment method was based on gradual referrals and inclusion criteria of the study. Each of the inpatient rooms was considered a cluster and then the samples were selected based on the list of random blocks from each of these rooms and were placed in the intervention and placebo groups. In the present study, the subjects and the statistical analyst were blinded. Blinding was done in the analysis section in such a way that by using data coding, the person who analyzed the data had no information about the allocation of the samples in each of the two study groups. leaven’s test applied for homogeneity of variance.

### Inclusion and exclusion criteria

The inclusion criteria were informed written consent; aged 20 to 80 years; having anxiety based on the Spielberger anxiety questionnaire (the minimum score obtained to enter the study was 4); no history of cardiomyopathy and lung, liver, and thyroid diseases; no chronic headaches, migraines, anxiety or mental disorders; no history of allergies, asthma, and Alzheimer; no opioid addiction; not taking anti-anxiety drugs that affect the nerves and psyche; no allergy to herbal medicines and perfumes; no history of cardiopulmonary resuscitation; no olfactory disorder; no death of relatives in the last month; full consciousness; no history of aromatherapy; and the ability to understand and speak Persian language. The exclusion criteria were unwillingness to continue the research, the occurrence of any allergic reaction, pain, shortness of breath, or sudden change in the patient’s hemodynamic status.

### Procedures

After having the written approval of the ethics committee of the Guilan University of Medical Sciences (IR.GUMS.REC.1399.482) and registration in the Iranian Clinical Trials Center (IRCT) under the number IRCT20080825001083N11 on 06/09/2020, recruitment was done over 4 months (January 20, 2020, to May 21, 2021). At first, a written consent form was obtained from all the samples, and then the demographic profile form and the STAI-6 questionnaire were completed by the researcher through an interview with the patient. Then, in both groups, the patient’s hemodynamic indices were measured half an hour before the intervention by the researcher. To ensure that the subjects were not allergic to chamomile essential oil, a skin allergy test was performed for the patients. In this way, a drop of chamomile essential oil was poured on the inner surface of the patient’s wrist and it was bandaged to prevent breathing it. After two minutes of skin contact, the contact site was observed, and if there were no signs of allergy (redness, hives, itching, etc.), the person entered the study [37]. In order to match the two groups, this was also done in the placebo group using a drop of sesame oil. The pure essential oil of the German chamomile plant with the scientific name Matricaria chamomilla was obtained from the Gorgan essential plant company [3, 4, 23]. The essential oil prepared at the Faculty of Pharmacy of GUMS was brought to a concentration of 10% using sesame oil [23]. Essential oils are diluted in vegetable oils [38], therefore, sesame oil was used in this study [23, 39, 40]. This oil is odourless and golden like other vegetable oils. It should be noted that so far no therapeutic, allergic [23] or anti-anxiety effects [39] have been reported through the inhalation of sesame oil and this oil does not affect chamomile essential oil [38]. None of the subjects had any skin allergy to chamomile. Then the intervention was carried out as follows: in the intervention group, according to the conventional treatment and care program of the department and the sleeping hours of the patients, 7 drops of chamomile essential oil of 10% were dripped on a sterile cotton ball with a dropper by the main researcher for two consecutive nights between 7 p.m. and 8 p.m [23]. Then the patients were asked to place the cotton in the palm at a distance of 5 cm from the nose [7] and take 10 deep breaths [4, 7]. Then the same cotton ball was placed inside a 70 cm gauze and tied around the patients’ neck similar to a necklace, and the next morning, after the end of the last 4 h, it was removed at 8 a.m [4]. In the placebo group, seven drops of sesame oil were replaced by this compound [23]. In both groups, the patient’s hemodynamic indices were measured four times in total by the researcher [7], half an hour before the intervention (19.30), one hour after the intervention [20], and at four-hour intervals until the next morning (4 a.m. and 8 a.m.) [26]. It should be noted that the patients in both groups received the usual care of the department. On the morning of the third day, after the end of the last 4 h at 8 a.m., once again, the Spielberger anxiety questionnaire was completed by the researcher through an interview with the patients.

### Data collection tools

The data collection tools consisted of three sections, including a demographic and medical characteristics questionnaire, the short form of the Spielberger State-Trait Inventory (STAI-6), and a form related to hemodynamic indices. The questionnaire of demographic and medical characteristics included gender, age, job, marital status, level of education, economic status, history of previous hospitalization, family history of heart disease, smoking history, and any underlying diseases. It was recorded through file review or an interview with the patient and family. The content validity of this questionnaire was confirmed by 10 faculty members of the Guilan University of Medical Sciences (GUMS). The STAI-6 questionnaire was prepared by Marteau & Bekker. Researchers have reported a reliability coefficient of ɑ=0.86 for this 6-item questionnaire [40]. In this questionnaire, there were several options for each item, including very low (1), low (2), medium (3), high (4), and very high (5). For items No. 1, 4, and 5, scoring was reversed as follows: very low (5), low (4), medium (3), high (2), and very high (1). Anxiety levels were classified based on these 6 items and after reversing the scores of those 3 items. Such that scores between 6 and 13 indicated weak anxiety, 14–22 moderate anxiety, and 23–30 severe anxiety. The minimum score obtained from this questionnaire was 6 and the maximum was 30. Abbaszadeh et al. (2018) and Bagheri-Nesami et al. (2014) in their studies to investigate anxiety in various disorders, evaluated the validity and reliability of the STAI-6 questionnaire, and the correlation coefficients between each of the scales were calculated to be 96% [41, 42]. The form related to hemodynamic indices was also designed by the researcher in such a way that these indices, including systolic blood pressure (SBP), diastolic blood pressure (DBP) and heart rate (HR) were recorded half an hour before, one hour after the intervention and at four-hour intervals for a total of four times after the intervention. In order to ensure stability in the measurement of indices, all measurements were done by the researcher.

### Statistical analysis

The Chi-square test was used to compare qualitative variables in both groups, and the t-test was used to compare quantitative variables such as age. Regarding the variables of anxiety and hemodynamic indices, the Kolmogorov-Smirnov test was first used to check the normality of the variables, and then, if they were normal, the independent t-test was used, and if they were not normal, the Mann-Whitney test was used. In order to calculate the trend of changes and also to compare the changes between the measurement times the Repeated Measures ANOVA test with Bonferroni, Grenhouse-Geisser test, Mauchly’s W test, and Sphericity Assumed test were used. Besides, MANOVA was used to determine the effect of aromatherapy on patients’ anxiety and hemodynamic indices. The significance level of the tests in this study was p˂0.05.

## Results

To test the normality of the distribution of quantitative data, the Shapiro-Wilk test was used. According to the results, the mean and standard deviation of the age of the subjects was equal to 58.2 ± 11.6, 59.7% of them were female, 70.8% lived in urban areas, 57.1% of them were housemakers, and 77.2% had a moderate economic status. The results of the present study indicated that both groups had no statistically significant differences and were homogeneous in terms of demographic characteristics (except gender, place of residence, employment and economic status) and medical characteristics (except the previous family history of hospitalization). Even though there was no significant difference between various age groups in the category related to age, the minimum effective dose of chamomile essential oil has been used according to the articles. Liver or kidney disease was one of the criteria for not entering the study, and no case of kidney or liver disease was reported. It should be noted that the results of the covariance test showed that the effects of statistically significant differences between the two groups in some demographic and medical characteristics (Gender, Place of residence, Employment status, Economic status, Hospitalization and Smoking history with *p*-value of 0.001, 0.021, 0.001, 0.036, 0.0123, 0.0174, respectively) that might have influenced the results of the study (effect of aromatherapy on anxiety level and hemodynamic indices) were neutralized.

The results of the present study showed that the distribution of anxiety scores before the intervention was the same in the two groups. However, the distribution of scores after the intervention and the changes in the anxiety score, before and after the intervention, between the two groups were significant (*p* < 0.001). Such that the mean and the median score after the intervention in the placebo group had higher values. The decrease in anxiety score after the intervention, in the intervention group, on average was (5.2 ± 1.9) with a median of 5 and in the placebo group on average was (1 ± 1.18) with a median of 1. Therefore, the level of anxiety of the patients was reduced after aromatherapy with chamomile essential oil.

Also, based on the results, there was no significant difference in the level of anxiety before the intervention between the intervention and placebo groups. Also, based on the results of the present study, there was no significant difference between the intervention and placebo groups before the intervention, but it became significant after the intervention (*p* < 0.001). The intensity of anxiety after the intervention in the intervention group was mild in most cases (62.3%), but it was (88.3% and 10.4%, respectively) moderate and severe in the placebo group. In general, the effect of the intervention on the state of anxiety in the intervention group was significant and reduced the level of anxiety, but no significant changes were observed in the placebo group (Table 1).

**Table 1:** Comparison of the level of anxiety before and after the intervention in the intervention and placebo groups

Anxiety		Group		
		Intervention	Placebo	Significance level*
		Numbers (%)	Numbers (%)	
Before the intervention	Mild	3 (3.90%)	0 (0.00%)	0.319*
	Moderate	63 (81.82%)	67 (87.01%)	
	Intense	11 (14.29%)	10 (12.99%)	
After the intervention	Mild	48 (62.34%)	1 (1.30%)	*<0.001
	Moderate	29 (37.66%)	68 (88.31%)	
	Intense	0 (0.00%)	8 (10.39%)	
	Significance level**	< 0.001	0.250	

*Fisher exact test **Sign test

The results indicated that the SBP and DBP levels on the first day before the intervention in both groups were statistically the same and not significant. However, after the intervention, it was significant in all hours 4, 8 and 12 during the first and second day (*p* < 0.05) in the intervention group. Also, the changes in HR on the first day before the intervention between the placebo and intervention groups were not statistically significant. However, after the intervention, it had a significant decrease in all hours 4, 8 and 12 during the first (*p* < 0.007) and the second days (*p* < 0.001) in the intervention group (Table 2).

**Table 2:** Comparison of average SBP, DBP and HR at different measurement times on the first and second days of the study in both groups

Variables	Groups				
	Intervention		Placebo		*P* value*
	Mean ± SD	Median	Mean ± SD	Median	
SBP time 1, day 1	126.36 ± 10.30	120.30	129.48 ± 14.81	130.00	0.202
SBP time 2, day 1	123.12 ± 13.59	120.00	129.16 ± 14.17	130.00	0.011
SBP time 3, day 1	119.9 ± 12.61	120.00	127.19 ± 13.24	120.00	< 0.001
SBP time 4, day 1	118.12 ± 12.11	120.00	127.66 ± 12.72	130.00	< 0.001
SBP time 5, day 1	116.43 ± 12.85	110.00	127.60 ± 12.66	130.00	< 0.001
SBP time 1, day 2	125.52 ± 12.73	120.00	131.10 ± 14.59	130.00	0.018
SBP time 2, day 2	120.06 ± 12.66	120.00	130.32 ± 14.63	130.00	< 0.001
SBP time 3, day 2	117.99 ± 11.65	120.00	129.87 ± 13.79	130.00	< 0.001
SBP time 4, day 2	116.62 ± 12.02	110.00	129.42 ± 12.93	130.00	< 0.001
SBP time 5, day 2	115.06 ± 12.02	110.00	129.16 ± 12.81	130.00	< 0.001
DBP time 1, day 1	80.56 ± 8.52	80.00	81.88 ± 7.35	80.00	0.352
DBP time 2, day 1	78.96 ± 8.12	80.00	81.75 ± 6.63	80.00	0.024
DBP time 3, day 1	76.30 ± 7.84	80.00	81.43 ± 6.78	80.00	< 0.001
DBP time 4, day 1	78.58 ± 7.86	80.00	81.62 ± 6.76	80.00	< 0.001
DBP time 5, day 1	74.74 ± 8.73	70.00	81.56 ± 6.65	80.00	< 0.001
DBP time 1, day 2	79.94 ± 8.01	80.00	83.31 ± 7.19	80.00	0.006
DBP time 2, day 2	77.01 ± 8.32	80.00	82.86 ± 7.27	80.00	< 0.001
DBP time 3, day 2	75.78 ± 8.23	80.00	82.73 ± 6.91	80.00	< 0.001
DBP time 4, day 2	74.29 ± 8.46	70.00	82.86 ± 6.51	80.00	< 0.001
DBP time 5, day 2	72.73 ± 8.90	70.00	82.73 ± 6.67	80.00	< 0.001
HR time 1, day 1	80.56 ± 5.89	80.00	81.82 ± 6.95	80.00	0.207
HR time 2, day 1	79.74 ± 5.64	80.00	81.43 ± 7.24	80.00	0.178
HR time 3, day 1	79.14 ± 5.72	80.00	81.48 ± 7.07	80.00	0.026
HR time 4, day 1	78.78 ± 5.65	78.00	81.58 ± 6.96	80.00	0.013
HR time 5, day 1	78.52 ± 5.40	78.00	81.56 ± 7.06	80.00	0.007
HR time 1, day 2	80.44 ± 5.63	80.00	82.18 ± 6.85	82.00	0.089
HR time 2, day 2	79.22 ± 5.83	80.00	82.09 ± 6.80	82.00	0.008
HR time 3, day 2	78.70 ± 5.93	78.00	82.04 ± 6.76	82.00	0.001
HR time 4, day 2	78.51 ± 5.78	78.00	82.03 ± 6.69	82.00	0.001
HR time 5, day 2	78.36 ± 5.76	78.00	81.99 ± 6.76	82.00	< 0.001

^*^RMANOVA

The results of the covariance test revealed the effect of aromatherapy on the level of anxiety, SBP, DBP and HR, after controlling for the intervening individual variables (gender, place of residence, job, economic status, and family history of heart disease) was clinically significant (*p* < 0.05). Therefore, the effect of statistically significant differences between the two groups of placebo and intervention in some demographic and medical characteristics that could influence the results of the study were neutralized (Table 3).

**Table 3:** RMANOVA results of the aromatherapy effect on anxiety, systolic and diastolic blood pressure and heart rate after controlling the effects of intervening variables

Source		Constant value	Group	Gender	Place of residence	Job	Economic status	Family history of heart disease
Significance level	SBP	0	0	0.591	0.795	0.915	0.635	0.261
	DBP	0	0	0.541	0.576	0.893	0.183	0.311
	HR	0	0.011	0.9	0.616	0.053	0.499	0.95
	Anxiety	0.301	0	0.546	0.346	0.081	0.404	0.424

## Discussion

The purpose of this research was to determine the effect of aromatherapy with chamomile essential oil on the anxiety and hemodynamic indices of ACS patients. The results revealed that aromatherapy with chamomile essential oil significantly reduced anxiety and adjusted hemodynamic indices in hospitalized ACS patients, which was consistent with the results of several studies. In the study of Gholami et al. (2018), investigating the effect of chamomile essential oil inhalation on the anxiety of endoscopy candidate patients, it was reported that the anxiety score changes after chamomile essential oil inhalation was significant in the intervention group. Therefore, chamomile essential oil inhalation was effective on patients’ anxiety before endoscopy and reduced it [23]. The study of Rahmani et al. similarly reported similar results. Such that in the group of ACS patients receiving aromatherapy with Lavender, Roman Chamomile and Naroli aromas, the mean score of anxiety was significantly reduced at the time intervals of one and 12 h after the intervention in both groups compared to before intervention [43]. In another study conducted by Rahnavardi et al., the scent of chamomile was effective on the anxiety level of nulliparous women and reduced it [44]. Also, Mohammad Aliha et al. investigated the effect of aromatherapy with lavender, chamomile and Citrus Aurantium on the vital signs of ACS patients. They reported that ANOVA with repeated measures showed a statistically significant decrease in HR, respiration, SBP and DBP in the aromatherapy group over time [8]. Also, in confirmation of this result of our study, Aalami et al. stated in their research that there was no significant difference between the mean anxiety scores before the intervention in the two groups. However, a significant difference was observed after the intervention. Also, there was a significant difference in the scores before and after the intervention between the two groups [4]. The study results of Zamanifar et al. were also in line with our study and a significant difference was observed in the anxiety scores before and after the intervention in the aromatherapy group [45].

In contrast to the result of this study, Donaldson et al. in their study, investigating the effect of aromatherapy with a combination of chamomile, sour orange, lemon, and orange essential oils on the anxiety experienced by clinical nurses, showed that no significant difference was observed between the mean of the pre-test and post-test anxiety scores for day and night shifts and all nurses [46]. Also, in the study of long-term treatment of generalized anxiety disorder by chamomile, Mao et al. reported that although people in the chamomile group experienced a reduction in the symptoms of generalized anxiety disorder, a decrease in mean arterial blood pressure, SBP and DBP, there was no difference in the change of HR between the groups [35]. These differences might be due to the difference in the type of essential oil used, the type and number of drops of essential oil, or the differences in the work method and tools, the research subjects, or the time of the intervention. Although the exact mechanism of the effectiveness of aromatherapy for anxiety is not yet known, scientifically it is believed that aromatherapy can be psychologically and physiologically effective [47]. The scent of volatile oils activates the olfactory nerve cells and thus stimulates the limbic system. Depending on the type of scent, nerve cells release different neurotransmitters. These neurotransmitters include enkephalin, endorphin, noradrenaline and serotonin. Due to the relationship between the olfactory sense and human emotions, perfumes could affect people psychologically and physically. Actually, scents could change emotions in humans [48, 49]. The above-mentioned studies have investigated the effect of chamomile by using different methods and in different applications, which confirm the sedative and anti-anxiety effects of chamomile. These results are clinically important in nursing care because improving these parameters without drugs is an important goal in healthcare, and doing so can reduce the complications caused by regular medical or therapeutic interventions.

The results of this study showed that aromatherapy with chamomile essential oil had an effect on reducing the anxiety of patients with ACS, which was significant compared to the control group. Also, reducing the anxiety can improve the hemodynamic indices. Then, aromatherapy as complementary therapy can be used as an independent nursing intervention to improve nurses’ performance. These findings are clinically important in nursing cares because improvement of these parameters without drugs is an important goal in care providing as doing so can reduce complications arising from regular medicinal or therapeutic interventions. Considering the important role of aromatherapy in reducing the anxiety of these patients as well as the high prevalence of this disease in all societies, administration of this inexpensive and simple nursing intervention can be an effective step in reducing the anxiety of patients and provide an opportunity for treatment team, particularly nurses, to provide better care.

### Limitations

Among the limitations of this research were the mental-psychological condition and personality characteristics of the samples, the subjectivity of the anxiety variable, family support, the hospital environment, and the relationship between perfumes with feelings and previous experiences of perfumes, which were beyond the control of the researcher.

## Conclusion

Application of aromatherapy as one of the methods of alternative medicine can decrease the anxiety and improve hemodynamic indices of acute coronary syndrome patients. Regarding the low side effects, non-aggressive and easy access to chamomile essential oils, the results of this study can be employed by the nurses of heart wards and coronary care unit (CCU) or the family of the mentioned patients to reduce their anxiety. Therefore, by using aromatherapy, an effective step can be taken to reduce treatment costs, side effects of drug treatments and increase the level of patient satisfaction. Also, the results of the current research can be a basis for future research by all the people who are involved in the care of patients and are interested in complementary and alternative treatments.

## Data Availability

The data that support the findings of this study are available from the corresponding author upon reasonable request.

## Abbreviations

ACS: Acute Coronary Syndrome
HR: Heart Rate
SBP: Systolic Blood Pressure
DBP: Diastolic Blood Pressure
ANOVA: Analysis of Variance
IHD: Ischemic Heart Diseases
WHO: World Health Organization
MI: Myocardial Infarction
CAM: Complementary and Alternative Medicine
STAI-6: Spielberger State-Trait Inventory
GUMS: Guilan University of Medical Sciences
CCU: Coronary Care Unit
